# The relationship between social support and depression among older adults with hypertension in urban communities: mediating effects of coping styles

**DOI:** 10.3389/fpsyt.2025.1508846

**Published:** 2025-02-11

**Authors:** Dan Li, Jin-Hua Jie, Hong Li, Xue-Mei Xia, Yilin Zhang, Yan Yang, Jianjun Xiang, Hai-Lin Zhuang

**Affiliations:** ^1^ School of Public Health and Health Management, Fujian Health College, Fuzhou, Fujian, China; ^2^ Department of Preventive Medicine, School of Public Health, Fujian Medical University, Fuzhou, Fujian, China; ^3^ Key Laboratory of Environment and Health, Fujian Province University, Fuzhou, Fujian, China; ^4^ School of Public Health, The University of Adelaide, Adelaide, SA, Australia

**Keywords:** hypertension, depression, older adult, social support, coping styles, mediating effects

## Abstract

**Background:**

Older adults with hypertension are at an increased risk of depression. Social support and coping style significantly influence this risk, with social cognitive theory suggesting that social support can affect an individual’s coping style, and coping style can affect the effective use of social support. However, the mediating role of coping style in the relationship between social support and depression in older hypertensive patients remains unclear. This study aimed to explore the inter-relationships between social support, coping style and depression in older hypertensive patients within a community setting, and to investigate the mediating effects of coping style.

**Methods:**

A cross-sectional questionnaire survey was conducted with 4211 older hypertensive patients from Fuzhou, Fujian Province, China. Participants were assessed for depression, social support, coping styles, and general demographic information. Pearson correlation analysis was employed to test the correlation between variables. Mediation effect test was conducted using SPSS macro program PROCESS with Bootstrap based Model 4, after controlling for confounding factors.

**Results:**

A total of 4211 valid questionnaires were collected, yielding an effective response rate of 98.6%. The average depression score among participants was 7.99 ± 4.92 points, with 29.5% of respondents experiencing varying degrees of depression. Both social support and coping style were associated with depression in older hypertensive patients. Specifically, objective support, subjective support, support utilization, and positive coping style were negatively correlated with depression, with correlation coefficients of -0.159, -0.160, -0.145, and -0.163, respectively. Conversely, negative coping style was positively correlated with depression, with a correlation coefficient of 0.170. Mediating effect analysis showed that coping style played a mediating role between social support and depression. Social support negatively moderated depression through positive coping style (moderating effect =-0.020, Bootstrap 95%CI [-0.027, -0.138], mediating effect value was 15.87%), while it positively influenced depression through negative coping style (moderating effect =0.012, Bootstrap 95%CI [0.008,0.017], intermediate effect value =9.52%).

**Conclusion:**

Social support exerts a direct negative effect on depression, while coping styles mediate the relationship between social support and depression. Interventions to reduce depression in this population should focus on enhancing social support (across objective, subjective, and utilization aspects) and promoting positive coping strategies, thus strengthening psychological resilience and improving overall health and quality of life for older adults with hypertension.

## 1 Introduction

China’s aging population is expanding rapidly, with individuals aged 60 and above reached 283.17 million in 2021, accounting for 18.95% of the total population (1). This demographic shift highlights the pressing need to address age-related health concerns, particularly chronic non-communicable diseases and mental health disorders, which have emerged as significant challenges (2). Evidence has shown that older people with chronic illnesses are more likely to experience depression (3). Hypertension, one of the most prevalent chronic diseases globally, affects over 1.2 billion individuals and represents a critical public health issue (4). In China, the number of older patients with hypertension has exceeded 160 million, with prevalence rates increasing with age, reaching as high as 70% among those over 95 (5). Hypertensive patients had a high prevalence of depression, with some studies reporting rates as high as 37.2% (6). As a negative emotional state, depression is not only a risk factor for hypertension (4) but also exacerbated by hypertension, significantly impairing both mental health and quality of life of hypertensive patients (7). Moreover, depressive symptoms were associated with poor blood pressure management, an increased risk of stroke and cardiovascular mortality, and poor prognosis (8).

Social support, including emotional experiences, feeling of respect, and support within society, has been identified as a crucial determinant of mental health outcomes. It can relieve stress and reduce the adverse impacts of stress on both physical and mental health (9). Many studies have consistently highlighted the role of social support in enhancing quality of life (10) and facilitating social adaptation, which in turn helps to mitigate negative emotional states (11, 12). Social support can be divided into three dimensions: objective support, subjective support, and support utilization. Objective support refers to visible or actual support, including direct material assistance or service forms of support. Subjective support relates to an individual’s emotional experience and satisfaction derived from feeling respected, supported, and understood within society. Support utilization reflects the extent to which individuals actively engage with and benefit from available support resources (13). Acquiring objective social support, fostering positive subjective emotional experiences, and effectively utilizing support are critical for improving individual mental health (14).

Coping styles refer to the cognitive and behavioral strategies that individuals employ to manage stress and adapt to challenging circumstances. These strategies can be broadly categorized into proactive approaches (e.g., solving problems and seeking assistance) and passive approaches (e.g., escaping, venting and tolerance). According to cognitive stress theory (15), coping styles affect well-being by influencing how people respond to stressful situations, and play a pivotal role in moderating the stress-depression relationship (16), with positive coping styles facilitating stress relief and exerting a negative predictive effect on depression, while negative coping styles are associated with higher levels of depressive symptoms (17, 18).

As an external environmental factor, social support can influence an individual’s mental health status interacting with internal factors such as coping styles (19). Social cognitive theory posits that individuals who perceive higher levels of social support are more likely to adopt positive coping behaviors, which can reduce the likelihood of depression (20). Similarly, Pearlin and Schooler (1978) (21) suggested that coping styles significantly influence a person’s ability to use available social resources. However, the extent to which different coping strategies mediate the relationship between social support and depression, particularly among older adults with hypertension, remains unclear. Understanding the magnitude and nature of these mediating effects is critical for developing effective interventions. While prior research has explored the association between social support and depression in older adults and those with chronic illnesses (22–24), limited studies have examined these relationships in the specific context of older hypertensive patients. Furthermore, the impact of coping styles on depression and mental health has been verified in general older people (25, 26) and socioeconomically disadvantaged older people (27), however, there is a lack of research that disaggregates social support into its three dimensions—objective support, subjective support, and support utilization—and analyzes their individual roles in relation to coping styles and depression. This gap is particularly evident when considering the dual roles of positive and negative coping styles as mediators.

This study aimed to investigate the interplay between social support, coping styles and depression among older hypertensive patients in urban communities, with a specific focus on the mediating role of coping styles between social support and depression. Findings of this study may provide both theoretical and empirical insights that inform early prevention and effective intervention for depression among older hypertensive patients.

## 2 Materials and methods

### 2.1 Study design and participant recruitment

The study was conducted in Fuzhou, the capital city of Fujian Province, located on the southeast coast of China. In 2020, people aged>60 years reached 19.1% of the population in Fuzhou. The prevalence of hypertension among adults in Fujian Province was 21.5%, with rates of 49.7% among those aged 60-69 and 66.9% among those aged 70 and above (28). Additionally, the prevalence of depression among adults in Fujian Province was reported to be 8.3%, with a rate of 15.0% for those aged 60 and above (29).

From March 2019 to June 2020, we conducted a cross-sectional questionnaire survey among older adults diagnosed with hypertension in Fuzhou. The inclusion criteria were: (1) individuals aged ≥60 years with a confirmed diagnosis of hypertension; (2) diagnosed by a secondary hospital or higher, or with a documented history of hypertension currently undergoing treatment; and (3) those who were aware of the purpose and agreed to participate in the survey. Exclusion criteria included: (1) individuals with mental disorders, dementia, and severe cognitive disorders; (2) those with communication difficulties impacting expression; and (3) individuals who refused participation.

Older hypertensive patients were approached under the support of local community health service centers when they carried out the National Basic Public Health Service (NBPHS) program for older hypertensive patients (30). The NBPHS program was initiated in 2009 to provide 14 categories of health services to all Chinese residents free of charge. NBPHSP covers the management of T2DM patients, including screening, regular follow-up, and health education. Local community health service centers and village clinics are responsible for the provision of NBPHSP services. A multi-stage cluster random sampling method was adopted to recruit older hypertensive patients in Fuzhou. First, two districts were randomly selected from the five urban districts of Fuzhou by drawing lots. Names of the five districts (Gulou, Taijiang, Cangshan, Jin ‘an, and Mawei) were put into a bowl and two names were randomly chosen. The two sampled districts (Taijiang District and Gulou District) have 22 community health service centers. Second, a unique number from 1 to 22 was allocated to the 22 community health service centers, and 11 of the 22 community health service centers were randomly selected as our research sites through an online random number generator (https://epitools.ausvet.com.au/randomnumbers). Participation is entirely voluntary, and no incentives were offered. Informed consent was obtained from participants before the survey. This study was approved by the Medical Ethics Committee of Fujian Health College.

### 2.2 Questionnaire design

The structured questionnaire consists of four parts. Part one collected demographic information, mainly including age, gender, education, marital status, average monthly income, smoking and drinking habits, exercise frequency, sedentary behavior, sleep patterns, eating habits, hobbies, living conditions, and family support. Part two is the Chinese version of the Geriatric Depression Scale (GDS), which contains 30 items (31). Each item is scored from 0 to 1, resulting in a total score ranging from 0 to 30. Higher scores indicate greater levels of depression. Scores from 0 to 10 suggest no depression, while scores of 11 or higher indicate varying degrees of depressive symptoms: 11-20 for mild depression and 21-30 for moderate to severe depression. The Cronbach’s α of GDS scale is 0.84. Part three is the Social Support Rating Scale (SSRS), adapted from the original scale (13). The scale consists of 10 items across three dimensions: subjective support, objective support, and support utilization. The higher the score, the better the level of self-perceived social support. The Cronbach’s α of SSRS scale is 0.77. Part four is the Simple Coping Style Questionnaire, which is a simple coping style scale developed by Jie (32). It includes 20 items divided into two sub-scales: positive coping (items 1-12) and negative coping (items 13-20). Responses were scored on a four-point scale (0-3), ranging from “never” to “often”. The Cronbach’s α of SCSQ scale is 0.85.

Following a pilot survey, the questionnaire was revised to ensure clarity and comprehensibility, with input from relevant experts. Prior to data collection, all investigators received standardized training to ensure consistency. The survey was conducted using a self-administered approach under the support from local community health service centers and CDCs (Center for Disease Control and Prevention). Faculty staff and students from the Department of Preventive Medicine, Fujian Health College were responsible for distributing the questionnaires, explaining the purpose of the study, and collecting the completed responses. Participants filled out the questionnaire independently, with on-site investigators available in the local community health service center for assistance as needed. Completed questionnaires were promptly reviewed and collected by the investigators on-site.

### 2.3 Statistical analysis

Data entry was performed using EpiData3.1. Statistical analyses were conducted using SPSS25.0 and Process4.1 macroprograms. Descriptive statistics for normally distributed measurement data were presented as mean ± standard deviation ( $\bar{\text{x}}$ ± s). Two independent samples t-tests were used for comparisons between two groups, while analysis of variance (ANOVA) was used for multiple group comparisons. LSD (the least significant difference) test was used for pairwise comparisons. Pearson correlation analysis was used to test the correlation between variables. Mediation effect analysis was conducted using Bootstrap-based model 4 via the SPSS macro program PROCESS. A *P-*value of <0.05 was considered statistically significant.

## 3 Results

### 3.1 Overall scores on depression, social support and coping styles

A total of 4211 valid questionnaires were collected in this study, with 2046 from Taijiang District and 2165 from Gulou District, resulting in an effective response rate of 98.6% (4211 out of 4270). The average GDS score was 7.99 ± 4.92, with 1240 participants (29.5%) meeting the criteria for depression. Among the depressed participants, 1203 cases (28.6%) exhibited mild depression, with an average GDS score of 13.78 ± 2.39. There were 37 participants (0.9%) were classified as experiencing moderate to severe depression, with an average GDS score of 22.70 ± 1.84. The total social support score averaged 38.52 ± 7.21, comprising an objective support score of 7.98 ± 2.58, a subjective support score of 23.44 ± 4.70, and a support availability score of 7.10 ± 2.11. The total score of coping style was 47.56 ± 9.28, with a positive coping score at 31.06 ± 6.56 and a negative coping score at 16.50 ± 4.80.

### 3.2 Comparison of scores on social support, coping styles and depression by different demographic variables

As shown in **Table 1**, significant differences in the depression scores were observed across different demographic characteristics, including age (*F*=18.14, *P*<0.001), education level (*F*=7.14, *P*=0.001), marital status (*t*=-2.10, *P*=0.036), average monthly income (*t*=4.95, *P*<0.001), drinking habit (*F*=3.39, *P*=0.034), and physical exercise engagement (*F*=53.71, *P*<0.001). Objective support also varied significantly by education level (*F*=5.98, *P*=0.003), marital status (*t*=6.58, *P*<0.001), living condition (*t*=12.85, *P*<0.001), and physical exercise engagement (*F*=11.68, *P*<0.001). Similarly, subjective support scores varied significantly across different age groups (*F*=14.17, *P*<0.001), education levels (*F*=13.94, *P*<0.001), marital status (*t*=9.54,*P*<0.001), living conditions (*t*=9.35, *P*<0.001), drinking habit (*F*=3.46, *P*=0.032), and physical exercise engagement (*F*=35.24, *P*<0.001). Notably, the utilization of social support showed significant differences by gender (*t*=-4.01, *P*<0.001), education level (*F*=6.18,*P*=0.002), living condition (*t*=2.08,*P*=0.038), smoking habit (*t*=2.52,*P*=0.012), and physical exercise engagement (*F*=15.92, *P*<0.001). The scores of positive coping style varied significantly by education level (*F*=8.54, *P*<0.001) and physical exercise engagement (*F*=28.35, *P*<0.001). Conversely, the scores of negative coping style demonstrated significant disparities across different age groups (*F*=5.55, *P*=0.004), average monthly income (*t*=2.15, *P*=0.031), and engagement in physical exercise (*F*=3.41, *P*=0.033).

**Table 1:** Scores on depression, social support, and coping styles among participants with different characteristics ( $\bar{x}$ ± *s*).

Variable		n	GDS score	Social support			Type of coping style	
				Objective support	Subjective support	Support utilization	Positive coping	Negative coping
Total		4211	7.99 ± 4.92	7.98 ± 2.58	23.44 ± 4.70	7.10 ± 2.11	31.06 ± 6.56	16.50 ± 4.80
**Gender**	Male	1771	7.82 ± 4.89	8.05 ± 2.64	23.55 ± 4.76	6.95 ± 2.07	31.09 ± 6.50	16.41 ± 4.75
	Female	2440	8.11 ± 4.93	7.93 ± 2.54	23.35 ± 4.66	7.21 ± 2.54	31.03 ± 6.60	16.57 ± 4.84
*t*/*P*			-1.84/0.066	1.44/0.150	1.318/0.187	**-4.01/<0.001**	0.30/0.764	-1.08/0.281
**Age (years)**	60~	1941	7.65 ± 4.75	8.07 ± 2.60	23.79 ± 4.69	7.08 ± 2.09	31.23 ± 6.62	16.48 ± 5.02
	70~	1643	7.99 ± 5.00	7.94 ± 2.55	23.31 ± 4.65	7.11 ± 2.14	30.88 ± 6.57	16.32 ± 4.67
	80~	627	9.01 ± 5.19	7.82 ± 2.61	22.68 ± 4.77	7.14 ± 2.07	30.99 ± 6.32	17.06 ± 4.43
*F*/*P*			**18.14/<0.001**	2.59/0.076	**14.17/<0.001**	0.22/0.806	1.33/0.265	**5.55/0.004**
**Education level**	Primary school and below	1204	8.41 ± 5.34	7.82 ± 2.52	22.89 ± 4.77	7.04 ± 2.16	30.65 ± 6.68	16.47 ± 4.64
	Junior high school	1359	7.95 ± 4.72	7.93 ± 2.53	23.44 ± 4.73	6.98 ± 2.05	30.80 ± 6.65	16.45 ± 4.91
	High school and above	1648	7.71 ± 4.73	8.15 ± 2.66	23.83 ± 4.58	7.24 ± 2.11	31.57 ± 6.36	16.56 ± 4.83
*F*/*P* value			**7.14/0.001**	**5.98/0.003**	**13.94/<0.001**	**6.18/0.002**	**8.54/<0.001**	0.24/0.787
**Marital status**	Married	3657	7.92 ± 4.84	8.09 ± 2.57	23.70 ± 4.62	7.09 ± 2.10	31.02 ± 6.50	16.46 ± 4.84
	Alone	554	8.43 ± 5.36	7.31 ± 2.54	21.68 ± 4.85	7.19 ± 2.17	31.29 ± 6.89	16.79 ± 4.54
*t*/*P* value			**-2.10/0.036**	**6.58/<0.001**	**9.54/<0.001**	-1.05/0.294	-0.88/0.379	-1.58/0.114
**Living condition**	Living alone	255	8.44 ± 5.18	5.79 ± 2.83	20.36 ± 5.47	6.80 ± 2.41	30.51 ± 6.97	16.20 ± 4.51
	Not living alone	3956	7.96 ± 4.90	8.12 ± 2.50	23.63 ± 4.58	7.12 ± 2.08	31.09 ± 6.53	16.52 ± 4.82
*t*/*P* value			-1.50/0.134	**12.85/<0.001**	**9.35/<0.001**	**2.08/0.038**	1.39/0.166	1.04/0.297
**Average monthly income**	<2000	981	8.67 ± 5.10	8.06 ± 2.47	23.23 ± 4.62	7.04 ± 2.03	31.39 ± 6.59	16.79 ± 4.88
	≥2000	3230	7.78 ± 4.84	7.96 ± 2.62	23.50 ± 4.72	7.12 ± 2.13	30.96 ± 6.54	16.41 ± 4.78
*t*/*P* value			**4.95/<0.001**	1.09/0.274	-1.58/0.115	-0.99/0.320	1.83/0.067	**2.15/0.031**
**Smoking**	yes	331	7.84 ± 4.80	7.88 ± 2.69	23.43 ± 4.76	6.82 ± 2.18	30.82 ± 7.08	16.65 ± 5.17
	no	3880	8.00 ± 4.93	7.99 ± 2.57	23.44 ± 4.70	7.12 ± 2.10	31.08 ± 6.51	16.49 ± 4.77
*t*/*P* value			0.57/0.568	0.75/0.457	0.02/0.986	**2.52/0.012**	0.63/0.527	-0.54/0.590
**Drinking**	often	216	8.18 ± 5.00	7.69 ± 2.54	23.80 ± 4.89	7.03 ± 2.22	30.96 ± 6.56	16.54 ± 5.02
	occasionally	342	7.33 ± 4.79	8.15 ± 2.87	23.99 ± 4.77	6.96 ± 2.11	30.60 ± 6.43	16.21 ± 4.63
	no	3653	8.04 ± 4.92	7.99 ± 2.56	23.36 ± 4.68	7.12 ± 2.10	31.11 ± 6.57	16.52 ± 4.81
*F*/*P* value			**3.39/0.034**	2.10/0.122	**3.46/0.032**	1.03/0.358	0.94/0.389	0.68/0.508
**Physical exercise engagement**	often	1821	7.21 ± 4.93	8.16 ± 2.65	23.98 ± 4.63	7.26 ± 2.22	31.89 ± 6.57	16.71 ± 4.98
	occasionally	1814	8.30 ± 4.62	7.93 ± 2.48	23.30 ± 4.57	7.06 ± 1.99	30.58 ± 6.17	16.40 ± 4.65
	no	576	9.47 ± 5.33	7.59 ± 2.63	22.15 ± 5.02	6.71 ± 2.01	29.93 ± 7.35	16.18 ± 4.67
*F*/*P* value			**53.71/<0.001**	**11.68/<0.001**	**35.24/<0.001**	**15.92/<0.001**	**28.35/<0.001**	**3.41/0.033**

Bold values are statistically significant at p<0.05.

### 3.3 Comparison of scores on social support and coping styles among participants with different levels of depression

As shown in **Table 2**, the scores for objective support, subjective support, support utilization, and positive coping style in the mild depression group were significantly lower than those in the normal group. Conversely, the scores for negative coping style were significantly higher in the mild depression group compared to the normal group. In the moderate to severe depression group, subjective support and support utilization scores were significantly lower than those in both the normal and mild depression groups. Additionally, the positive coping style score was significantly lower in the moderate to severe depression group compared to the normal group. Conversely, the negative coping style score was higher in the moderate to severe depression group compared to the normal group.

**Table 2:** Scores of social support and coping styles among participants in different depression groups ( $\bar{x}$ ± *s*, score).

Group	n	GDS score	Social support			Coping style	
			Objective support	Subjective support	Support utilization	Positive coping	Negative coping
Normal group	2971	5.46 ± 3.04	8.20 ± 2.62	23.83 ± 4.64	7.25 ± 2.17	31.57 ± 6.62	16.08 ± 4.76
Mild depression group	1203	13.78 ± 2.39^a^	7.47 ± 2.42^a^	22.57 ± 4.68^a^	6.77 ± 1.90^a^	29.89 ± 6.23^a^	17.45 ± 4.80^a^
Moderate to severe depression group	37	22.70 ± 1.84^a,b^	7.62 ± 2.40	19.81 ± 5.03^a,b^	5.89 ± 2.00^a,b^	28.05 ± 6.01^a^	18.97 ± 3.05^a^

“a” means compared with the normal group and *P*<0.05; “b” means compared with the mild depression group and *P*<0.05.

### 3.4 Correlation between subjects’ social support, coping styles and depression

Correlation analysis was conducted to examine the relationship between depression scores and various factors including objective support, subjective support, supportive utilization, positive coping style and negative coping style. Significant negative correlations were observed between depression scores and objective support, subjective support, support utilization, and positive coping style. Conversely, depression scores exhibited a positive correlation with negative coping style, as shown in **Table 3**.

**Table 3:** Correlation between social support, coping styles and depression.

	GDS score	Objective support	Subjective support	Support utilization	Positive coping	Negative coping
GDS score	1.000					
Objective support	-0.159^a^	1.000				
Subjective support	-0.160^a^	0.379^a^	1.000			
Support utilization	-0.145^a^	0.267^a^	0.337^a^	1.000		
Positive coping	-0.163^a^	0.171^a^	0.245^a^	0.217^a^	1.000	
negative coping	0.170^a^	-0.104^a^	-0.062^a^	-0.055^a^	-0.318^a^	1.000

“a” indicates a statistical significance(*P*<0.001).

### 3.5 Mediating effects of coping style between social support and depression in older hypertensive patients

**Figure 1:**
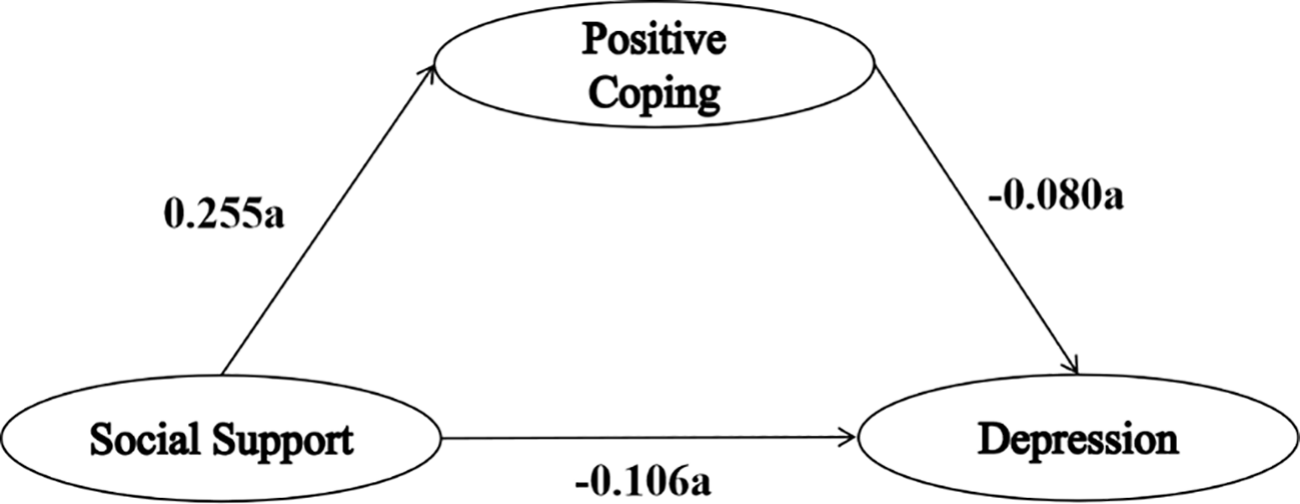
The mediating effect of positive coping style on social support and depression. “a” indicates statistical significance (*P*<0.001).

**Figure 2:**
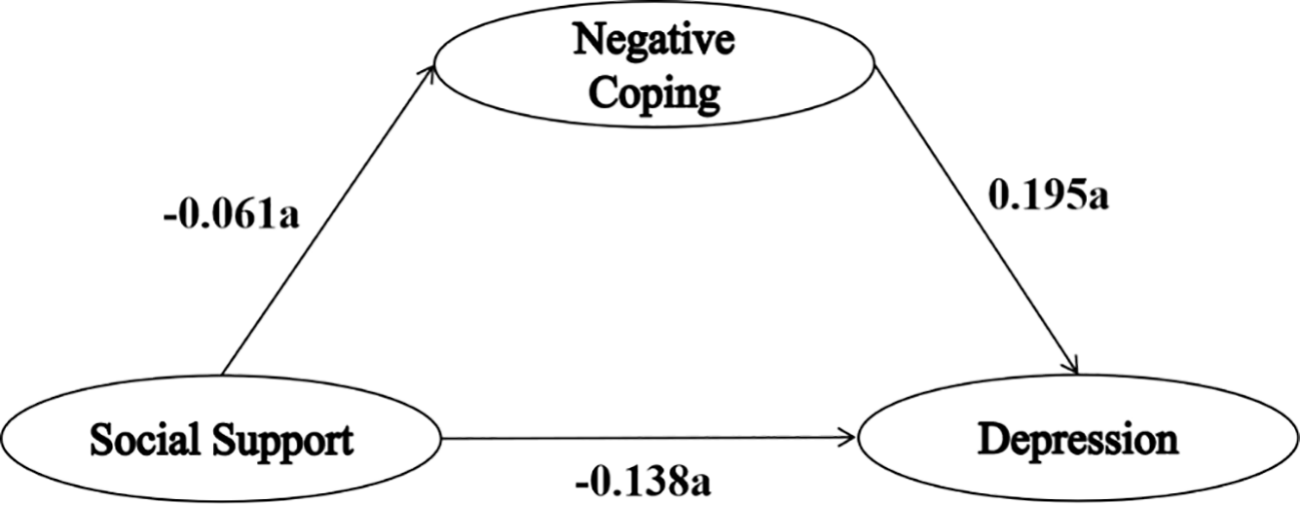
The mediating effect of negative coping style between social support and depression. “a” indicates statistical significance (*P*<0.001).

As shown in **Table 4**; **Figures 1**, **2**, model 4 of the SPSS macro program PROCESS was used to investigate the mediating role of coping style, with social support as the independent variable, depression as the dependent variable, and positive and negative coping styles as the mediating variables, respectively. The mediation analysis included age, education, marital status, living conditions, alcohol consumption, average monthly income, and physical exercise as control variables.

**Table 4:** Verification of mediating effects model of coping style.

Regression equation		Fitting index			Coefficient significance		
Result variables	Predictive variables	R	R^2^	F value	B value	t value	95%CI
Depression	Social support	0.263	0.069	34.697^a^	-0.132	-12.170^a^	-0.153,-0.110
Positive coping	Social support	0.306	0.094	54.354^a^	0.255	18.493^a^	0.228,0.282
Negative coping	Social support	0.116	0.013	7.119^a^	-0.061	-5.825^a^	-0.082,-0.041
Depression	Positive coping	0.280	0.079	39.819^a^	-0.080	-6.855^a^	-0.103,-0.057
Depression	Negative coping	0.322	0.104	54.139^a^	0.195	12.925^a^	0.165,0.224

“a” indicates statistical significance(*P*<0.001); All the models were tested by controlling the variables of age, education, marital status, living condition, alcohol consumption, average monthly income and physical exercise.

The regression coefficient for social support on depression was -0.132, which adjusted to -0.126 after adding coping style into the mediation effect model. Despite a reduction in the regression coefficient, it remained statistically significant. At the same time, social support had a positive predictive effect on positive coping styles (B value=0.255), and positive coping styles had a negative predictive effect on depression (B value=-0.080). Similarly, social support had a negative predictive effect on negative coping styles (B value=-0.061), and negative coping styles had a positive predictive effect on depression (B value=0.195).

As shown in **Table 5**, the results of the mediation effect analysis showed that the 95% confidence interval of each path did not include 0, indicating significant mediation effects of both positive and negative coping styles. In the mediating effect model of positive coping style, the direct effect of social support on depression was -0.106, with a mediating effect attributed to positive coping style of -0.020, representing 15.87% of the total effect. Conversely, in the mediating effect model of negative coping style, the direct effect of social support on depression was -0.138, accompanied by a mediating effect attributable to negative coping style of 0.012, constituting 9.52% of the total effect.

**Table 5:** Analysis of the mediating effects of coping style on social support and depression.

Model	Path	*B*	*Bootstrap SE*	Bootstrap 95%CI	Mediating effects value(%)
	Total effects of social support on depression	-0.126	0.011	-0.147, -0.106	
Positive coping model	Direct effects of social support on depression	-0.106	0.011	-0.127, -0.085	
	Mediating effects of positive coping	-0.020	0.003	-0.027, -0.138	15.87%
Negative coping model	Direct effects of social support on depression	-0.138	0.010	-0.159, -0.118	
	Mediating effects of negative coping	0.012	0.002	0.008, 0.017	9.52%

The model has adjusted for age, education, marital status, living conditions, alcohol consumption, average monthly income, and physical exercise for analysis.

## 4 Discussion

This study reveals a concerningly high prevalence of comorbid hypertension and depression among older adults in the communities of Fuzhou City probably due to a rapidly aging population, highlighting the critical need for public health interventions aimed at reducing depressive symptoms in this vulnerable population. The 29.5% prevalence of depression observed in hypertensive older adults in this study is higher than the 26.8% reported by Li et al. (2015) in a meta-analysis involving 30,796 samples (33). In comparison to national and international benchmarks, this rate is significantly higher than the national average in China (28.5%) and considerably exceeds the rates observed in countries like Brazil (12.1%), the Netherlands (11.4%), Ghana (10.5%), and Norway (6.2%) (34). These findings emphasize the complex interplay between physical and psychological factors, which intensify the condition of older hypertensive adults. The association between hypertension and depression is multifaceted. Chronic diseases like hypertension not only contribute to psychological stress, which can lead to depression, but depression also worsens hypertension through physiological mechanisms like elevated cortisol levels. Thus, managing both conditions in an integrated manner is imperative.

Our study further underscores the importance of social support in mitigating depression in older hypertensive individuals. We observed a negative association between social support and depression, indicating that increasing social support can significantly reduce the likelihood of depression. This aligns with previous research on older adults and individuals with chronic illnesses (22–24). By disaggregating social support into three dimensions (objective support, subjective support, and support utilization), we were able to explore the specific contributions of each. Our findings show that all three dimensions of social support are inversely related to depression. Specifically, objective support, such as assistance with daily tasks or financial aid, alleviates stress, while subjective support, reflecting emotional experiences of care and respect, enhances psychological resilience. Additionally, support utilization, which refers to an individual’s active engagement with available resources, plays a key role in reducing depressive symptoms. This highlights the multifaceted nature of social support, where both material assistance and emotional connections are essential in combating depression.

The aging process often brings challenges such as reduced social opportunities, loss of social roles, decline of physical function, absolute and relative scarcity of material and mental resources (35), as well as hypertension symptoms such as headache, dizziness, and insomnia, increase the risk of depression among older adults. Community health service workers may need to pay more attention to improving the skills of older adults with hypertension to prevent depression. Teaching individuals how to confide in others and actively seek help could empower them to reduce the occurrence of depression. Moreover, health education programs aimed at family members are vital for increasing awareness of depression in older individuals. Strengthening the support networks within families, such as enhancing emotional care and practical assistance from children and relatives, can provide significant protection against depression. Encouraging active social participation through community engagement programs also plays a pivotal role in reducing depression risk (36). It is essential for the government to utilize grassroots organizations, such as senior recreation centers, elderly associations, and home-based care services, to facilitate social participation and meet the psychosocial needs of older adults, thereby buffering the negative effects of aging and chronic illness.

Our findings underscore the significant role of coping styles in shaping the relationship between social support and depression. Specifically, positive coping styles, such as problem-solving and seeking emotional support, were found to be negatively correlated with depression. These strategies enable individuals to actively address stressors and leverage social resources more effectively, which mitigates the psychological burden and reduces depressive symptoms. Conversely, negative coping styles, such as avoidance and self-blame, were positively correlated with depressive symptoms. These maladaptive strategies often exacerbate feelings of helplessness and isolation, thereby intensifying depressive symptoms. These results align with cognitive stress theory, which posits that coping strategies serve as mediators that influence psychological outcomes in response to stress (37). Moreover, our findings highlight the dual role of coping styles—not only as mediators but also as modulators of an individual’s ability to utilize social support. Positive coping styles enhance the capacity to draw upon social resources, fostering stronger emotional resilience and reinforcing the protective effects of social support against depression. In contrast, negative coping styles undermine the effectiveness of social support by impeding meaningful engagement with social networks or fostering self-perceptions of inadequacy and failure (21).

Through mediation effect model analysis, we found that social support directly and significantly alleviates depression. Positive coping styles enhanced this protective effect by amplifying the regulatory influence of social support on depression, whereas negative coping styles weakened it. Specifically, the instrumental, practical aspects of social support, such as providing tangible assistance, exhibited a direct negative association with depressive symptoms. However, experiential and emotional aspects of support revealed varied effects depending on the coping strategies employed. These findings are consistent with the study by Dongling Liu et al. (38), which highlighted the moderating role of coping styles in the relationship between social support and depression among Chinese adolescents. Furthermore, our analysis showed that social support positively predicts adaptive coping strategies, such as seeking help and problem-solving, while negatively predicting maladaptive strategies, such as avoidance and self-blame. Older hypertensive individuals with higher levels of social support are more inclined to adopt positive coping strategies, aligning with evidence from studies on empty-nest older adults, high school students, and vocational students (39–41).

In addition to direct effects of social support on depression, we found that coping styles mediated these associations to a significant degree. In our modeling, 15.87% of the association between social support and depression was mediated by positive coping style, and 9.52% by negative coping styles, in line with findings from Irena Milaniak (42) Lijuan Chen (25) and Hadi Zamanian (43). Although domestic and international scholars have different definitions and interpretations on coping style, it is widely recognized as a strategy and method to alleviate the impact of stressful events on individuals. Coping styles are influenced by various factors such as personality traits (44), family upbringing style, and age (40). Notably, coping styles tend to exhibit relative stability in older adults. Therefore, enhancing social support, particularly through objective forms support, such as economic assistance, medical services, and guidance on healthy living, may help mitigate the psychological impact of diseases and life stressors on older hypertensive patients, who often rely on negative coping strategies. Although coping styles are influenced by personality, they are behavioral activities that can evolve with experience (45, 46). Furthermore, increasing older adults’ participation in physical exercise (47), improving their living conditions, and managing chronic diseases (48) can promote the use of positive coping strategies in response to high-stress situations, ultimately benefiting both their physical and mental health.

Several limitations in this study should be acknowledged. First, the cross-sectional design of the study limits our ability to establish causal relationships among social support, coping styles, and depression. The temporal ordering of these variables remains uncertain, and longitudinal or experimental studies are needed to clarify these relationships. Second, although our study included a large sample of older adults with hypertension from urban communities in Fuzhou, the findings may not be generalizable to rural populations or other demographic groups. Third, the use of self-reported questionnaires may introduce reporting bias, such as social desirability or recall bias, which could affect the accuracy of the data. Fourth, while we controlled several covariates in the analysis, other factors (e.g., personality traits or life events) might also influence the relationships between social support, coping styles, and depression but were not accounted for in this study.

## 5 Conclusion

The growing population of older adults with hypertension, coupled with the rapid aging of China’s population, represents a significant public health challenge. This study underscored the critical role of social support, not only in alleviating depressive symptoms but also in shaping coping mechanisms that help older adults manage stress. Current social policies predominantly focus on improving the physical environment for older people; however, this study highlights the need for more comprehensive strategies that enhance social support networks and promote positive coping mechanisms. Strengthening psychological resilience through targeted social and psychological interventions can significantly improve both the physical and mental health of older people with chronic diseases. Future public health measures should prioritize fostering social connections, providing psychological support, and empowering older adults to better manage their health, thereby improving their quality of life and reducing the burden of chronic diseases.

## Data Availability

The original contributions presented in the study are included in the article/supplementary material. Further inquiries can be directed to the corresponding authors.
